# A Surface Dielectric Barrier Discharge Plasma for Preparing Cotton-Fabric-Supported Silver Nanoparticles

**DOI:** 10.3390/nano9070961

**Published:** 2019-07-01

**Authors:** Zhiyuan Fan, Lanbo Di, Xiuling Zhang, Hongyang Wang

**Affiliations:** College of Physical Science and Technology, Dalian University, Dalian 116622, China

**Keywords:** surface DBD, atmospheric-pressurecold plasma, cotton fabric, silver, antimicrobial

## Abstract

Cotton-fabric-supported silver nanoparticles (Ag NPs) have aroused great attention due to their remarkable physical and chemical properties and excellent broad-spectrum antibacterial performance.In this work, a surface dielectric barrier discharge (DBD) plasma method is developed and employed to prepare cotton fabric supported Ag NPs (Ag/cotton) for the first time. UV-Vis and X-ray photoelectron spectroscopy (XPS) results confirm the formation of Ag NPs. TEM images show that the size of Ag NPs is in the range 4.8–5.3 nm. Heat-sensitive cotton fabrics are not destroyed by surface DBD plasma according to FTIR and XRDresults. Wash fastness of the Ag/cotton samples is investigated using ultrasonic treatment for 30 min and it is shown that the Ag NPs possess good adhesion to the cotton fabric according to UV-Vis spectra. Antibacterial activity of the Ag/cotton samples shows that obvious bacteriostasis loops are observed around the samples with the appearance of both Gram-negative bacterium *Escherichia coli* (*E. coli*) and Gram-positive bacterium *Bacillus subtilis* (*B. subtilis*). The average diameter of the bacteriostasis loops against both *E. coli* and *B. subtilis* becomes larger with an increasing silver loading amount.This work provides a universal, fast, simple, and environmentally-friendly cold plasma method for synthesizing Ag NPs on heat-sensitive materials at atmospheric pressure.

## 1. Introduction

Cotton fabrics are highly popular due to their excellent properties such as comfortability, air permeability, flexibility, bio-degradable ability, and good absorption of water. However, they provide ideal conditions (such as humidity, temperature, and nutrients, etc.) for bacteria and fungi to grow due to their high specific surface areas. In cotton fabrics, therefore, pathogens breed quickly, send out an unpleasant odor, and lead to textile decoloration and value shrinking, which accelerates the cross-spread of infection and aggravates human health. To solve these problems, great efforts have been devoted to modifying cotton fabrics by adding various antibacterial chemicals (including silver, copper, tungsten carbide, graphene derivatives, and tellurium, etc.) to improve their antibacterial activity [1,2,3,4,5]. Among these antibacterial chemicals, silver is widely used due to its low cost, and silver nanoparticles (Ag NPs) generally exhibit remarkable physical and chemical properties and excellent antibacterial performance against a wide range of pathogens [6].

According to the reduction process of silver ions into Ag NPs, the methods for preparing cotton-fabric-supported silver nanocomposites can be categorized into an exsitu reduction method and an insitu reduction method. With regards to theexsitu reduction method, an aqueous colloidal solution of Ag NPs is first prepared and then is supported on cotton fabrics by functional groups [7,8]. For example, Xu et al. [9] prepared antimicrobial cotton fabric by a one-pot modification process using a colloidal solution of Ag NPs via carboxymethyl chitosan (CMC), which was used as a binder and stabilizer. Consequently, the Ag NPs were uniformly distributed on the cotton fabric surface and exhibited remarkable and durable antibacterial activity. In addition, pressurized gyration has been proven to be an efficient exsitu method for mass production of polymeric fibers with antibacterial NPs [10,11]. Details about the method can be found in a feature article written by Edirisinghe et al. [12]. In the case of the insitu reduction method, silver ions arefirst supported on cotton fabric and then reduced by chemical reagents to metallic Ag NPs [13,14]. For example, El-Naggar et al. [15] activated loomstate, scoured, and bleached cotton fabrics by ethanolamine treatment and obtained three fabric-supported Ag NPs with ethanolamine in the absence of another external precursor. The size of the Ag NPs increased with increasing AgNO_3_ concentration. The loomstate- and scoured-cotton-fabric-supported Ag NPs exhibited excellent durability and antibacterial activity. As mentioned above, both the exsitu method and insitu method have prepared cotton-fabric-supported Ag NPs with high antibacterial activity and durability. However, excess chemical reagents are generally required to be served as reducing agents. Therefore, more attention has been paid to environmentally-friendly preparation methods, such as the bio-synthesis method [16], photo-reduction method [17], and cold plasma method [1].

Cold plasma is characterized by a high electron temperature and low gas temperature (which can be close to room temperature). It has been proven to be an environmentally-friendly and fast method for reducing metal ions to synthesize supported metal catalysts due to its non-thermal equilibrium properties [18,19]. The synthesized supported metal catalysts possess small and high dispersion metal NPs [20,21] and an enhanced metal-support interaction due to the strong electric field in plasma [22,23,24]. Hence, synthesized metal catalysts generally exhibit high catalytic activity and stability. Li et al. [25] prepared cotton-fabric-supported Ag NPs by room temperature cold plasma reduction at low pressure and the samples exhibited high antibacterial activity against the bacteria *E. coli* and *B. subtilis*. Compared to low-pressure (LP) cold plasma, atmospheric-pressure (AP) cold plasma, which does not require high-cost and sophisticated vacuum equipment, is more attractive. However, since the electron temperature of AP cold plasma is generally low at atmospheric pressure, hydrogen-containing species (H_2_, CH_4_, NH_3_, and ethanol, etc.) should be used as the reducing agents [26,27,28]. Consequently, the generally adopted AP cold plasma jet, plate-to-plate, and coaxial dielectric barrier discharges(DBD) are not suitable for preparing cotton-fabric-supported Ag NPs due to the open-air circumstances or the direct contact between the heat-sensitive cotton fabrics and the discharge zone.

Surface DBD is one important type of DBD discharge in which the discharges occur between the dielectric and discharge electrodes. It has been widely used in aerodynamic flow control [29], seed treating [30], film deposition [31], ozone production [32], and bacteria sterilization [33], etc. For materials treatment, surface DBD may allow any substrate thickness and is promising for large scale treatment. In this work, a surface DBD plasma was employed to reduce silver ions to prepare cotton-fabric-supported Ag NPs at atmospheric pressure for the first time. The heat-sensitive cotton fabric is not closely contacted with the discharge zone but is placed below it at a 4 mm distance. Hence, the generated active hydrogen species in surface DBD plasma is able to reduce the silver ions supported on cotton fabric into metallic Ag NPs without destroying thecotton fabric structure. The prepared samples exhibit high antibacterial activity against both the Gram-negative bacterium *E. coli* and the Gram-positive bacterium *B. subtilis*.

## 2. Materials and Methods

### 2.1. Preparation of the Ag/Cotton Samples

Prior to preparing the cotton-fabric-supported silver (Ag/cotton) samples, 100% plain-woven cotton fabrics (320 g·m^−2^) were cut into small pieces (2.5 × 2.5 cm^2^) and cleaned by Triton X-100 at 60 °C for 1 h. Silver nitrate (AgNO_3_) and polyvinyl pyrrolidone (PVP, molecular weight = 1000–1,300,000) were purchased from Kemiou Chemical Reagent Co., Ltd. (Tianjin, China). Ag/cotton samples were prepared by impregnation, which was followed by cold plasma treatment. Typically, a piece of cotton fabric was put into the AgNO_3_ aqueous solution and PVP and kept in the dark for 2 h. Then, the wet samples were centrifuged at 6000 rpm for 10 min to remove the excess AgNO_3_. Afterwards, the samples were flattened and put into asurface DBD reactor.

**Figure 1:**
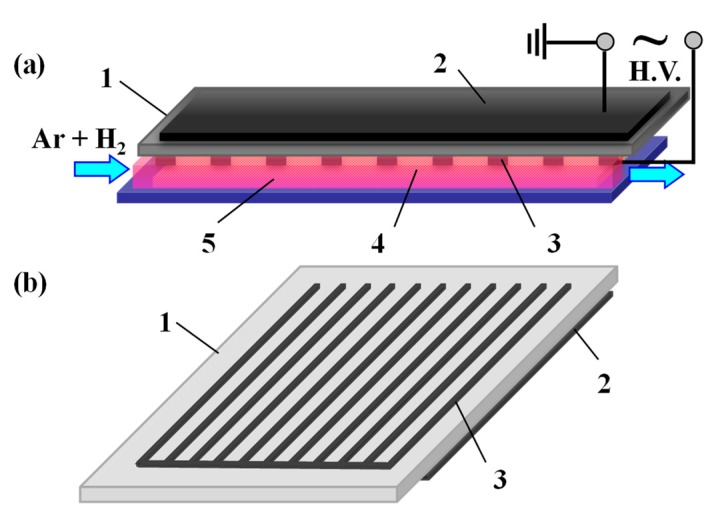
(**a**) Schematic diagram of the surface dielectric barrier discharge (DBD) device for preparing Ag/cotton samples and (**b**) the electrode pattern for the surface DBD. 1: alumina plate, 2: induction electrode, 3: discharge electrode, 4: Ag/cotton sample, 5: cold plasma.

Figure 1 illustrates a schematic diagram of the surface DBD device used in preparing the Ag/cotton samples, as well as the electrode pattern for the surface DBD. The surface DBD electrode system consisted of a comb-like discharge electrode and an induction electrode separated by an alumina plate with 1 mm thickness. Both the discharge electrode and induction electrode were made of tungsten. The discharge electrode comprised 11 stripes (1 mm wide and 4 mm strip-to-strip) connected all together at the end and covering 9.0 × 5.0 cm^2^ in area. The induction electrode was a 7.6 × 4.2 cm^2^ rectangle. A mixture of high purity Ar and H_2_ (>99.999%) was adopted as the working gas and the generated active hydrogen species (H, H^*^, H_2_^*^) were used to reduce the silver ions into Ag NPs [11,12]. The total flow rate of the working gas was 100 mL·min^−1^ and the gas ratio of Ar to H_2_ was 1:1. A CTP-2000K power supply (Nanjing Suman Electronic Co. Ltd., Nanjing, China) was employed to ignite the surface DBD electrode system to generate AP cold plasma. The discharge voltage was a 5.7 kV peak-to-peak sinusoidal high voltage and the discharge frequency was kept at 10.4 kHz. The Ag/cotton samples were placed below the surface DBD electrode system at a 4 mm distance. The treatment was performed three times, taking 2 min for each treatment. After surface DBD plasma treatment, the as-prepared Ag/cotton samples were dried at 120 °C for 2 h under vacuum in the dark. Then, they were rinsed with deionized water to get rid of the residual water-soluble AgNO_3_ salt and dried under vacuum for another 2 h. Various AgNO_3_ concentrations (1, 2, 5, and 10 mM·L^−1^) were adopted, while the PVP concentration was kept constant at 10 mg·ml^−1^. Accordingly, the Ag/cotton samples prepared by surface DBD plasma were marked as Ag/Cotton-1, Ag/Cotton-2, Ag/Cotton-5, and Ag/Cotton-10, respectively.

### 2.2. Characterization

X-ray photoelectron spectroscopy (XPS) experiments were performed on a Thermo Fisher Scientific K-Alpha XPS spectrometer (Waltham, MA, USA) to investigate the valence state of the silver element. The binding energies were calibrated using a C1s peak at 284.6 eV as the reference. Infrared spectra of the samples were recorded on a Thermo Scientific Nicolet 6700 Fourier Transform Infrared Spectrometer (Waltham, MA, USA) using attenuated total reflection in the region of 6000–600 cm^−1^ with a resolution of 4 cm^−1^ by accumulating 32 scans. UV-Vis diffuse reflectance spectra (DRS) of the samples were collected for the spectral range 200–800 nm using a Hitachi U-3900 UV-Vis spectrometer (Tokyo, Japan) with BaSO_4_ as a reference. XRD data of the samples were examined via a Dandong Haoyuan DX-2700 X-ray diffractometer (Dandong, China) using CuK_α1_ radiation (λ = 1.54178 Å). The morphology of the samples was examined using a Zeiss Sigma500 SEM (Jena, Germany) at a 5 kV accelerating voltage. Prior to SEM investigation, the cotton fabric samples were coated with a thin film of gold by sputtering. The SEM images were recorded at magnifications of 5000× and 10,000×. TEM and high resolution TEM (HRTEM) experiments were carried out using a FEI Tecnai G2 F20 S-Twin TEM (Hillsboro, OR, USA). Prior to testing, the samples were cut into pieces and placed in ethanol with ultrasonic dispersion for 120 min. Then, a few drops of the suspension were supported on an ultra-thin carbon film and dried in air at room temperature. The sizes and size distribution of the Ag NPs were obtained by calculating sizes for more than 300 Ag NPs.

### 2.3. Wash Fastness

Wash fastness of the Ag/cotton samples was estimated by ultrasonic leaching method. Typically, a piece of Ag/cotton sample (2.5 × 2.5 cm^2^) was immersed in a Pyrex beaker with 30 mL of deionized water. Then, the beaker was put into a KQ2200DB digital control ultrasonic generator (Kunshan Ultrasonic Instrument Co. Ltd., Kunshan, China) with a frequency of 40 kHz and an ultrasonic input power of 100 W. Ultrasonic leaching was performed for 30 min at a 30 °C sonication bath temperature. Then, the samples were dried under vacuum at 120 °C for 2 h. The wash fastness of the samples was tested by comparing their UV-Vis DRS spectra before and after ultrasonic leaching treatment.

### 2.4. Antibacterial Activity

The antibacterial activity of the Ag/Cotton samples against the Gram-negative bacterium *E. coli* and the Gram-positive bacterium *B. Subtilis* (Shanghai Luwei Technology Co. Ltd., Shanghai, China) was evaluated by the disk diffusion method and dilution method of plate counting. The process was similar to that used in a previous work [25]. Mueller-Hinton (MH) agar medium (6 g∙L^−1^ beef powder, 1.5 g∙L^−1^ soluble starch, 17.5 g∙L^−1^ acid hydrolyzed complex protein, and 17 g∙L^−1^ agar), deionized water, and the petridish were autoclaved prior to use. To compare antibacterial activity, blank cotton fabric and the Ag/cotton samples were cut into pieces of equal size (1.0 × 1.0 cm^2^). The *E. coli* and *B. subtilis* bacteria were cultured with MH agar medium in a constant temperature incubator at 37 °C for 8 h, respectively. Afterwards, the bacteria cultures were diluted (*E. coli*: folds 100, ~10^7^ CFU∙mL^−1^; *B. subtilis*: folds 100, ~10^7^ CFU∙mL^−1^. CFU is cell colony forming unit) with deionized water and MH agar, and the inoculums were evenly spread over the petridish, respectively. Lastly, the samples were planted onto the agar plates and incubated at 37 °C for 24 h to test their antibacterial activity by examining the diameter of the growth-inhibition zone.

## 3. Results and Discussion

**Figure 2:**
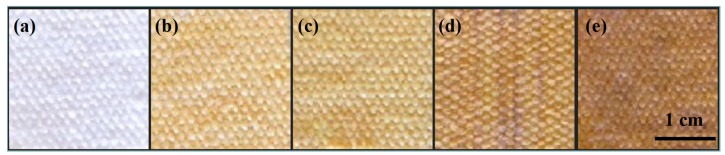
Photographs of (**a**) blank cotton, (**b**) Ag/Cotton-1, (**c**) Ag/Cotton-2, (**d**) Ag/Cotton-5, and (**e**) Ag/Cotton-10.

Figure 2 shows the photographs of the blank cotton and the surface DBD plasma-prepared Ag/cotton samples with different silver loading amounts. Clearly, the color of the Ag/cotton samples has changed from colorless to yellow following surface DBD plasma treatment; this is generally thought to be the color of metallic Ag NPs. This change reveals that the silver ions in AgNO_3_ can be reduced to metallic silver by surface DBD plasma. Moreover, by increasing the loading amount of silver, the color of the Ag/cotton samples changes from light yellow to brownish yellow, indicating that more Ag NPs are formed at a high silver loading amount. Similar phenomena were also observed in the preparation of Ag/cotton samples by DC glow discharge cold plasma at low pressure [25].

**Figure 3:**
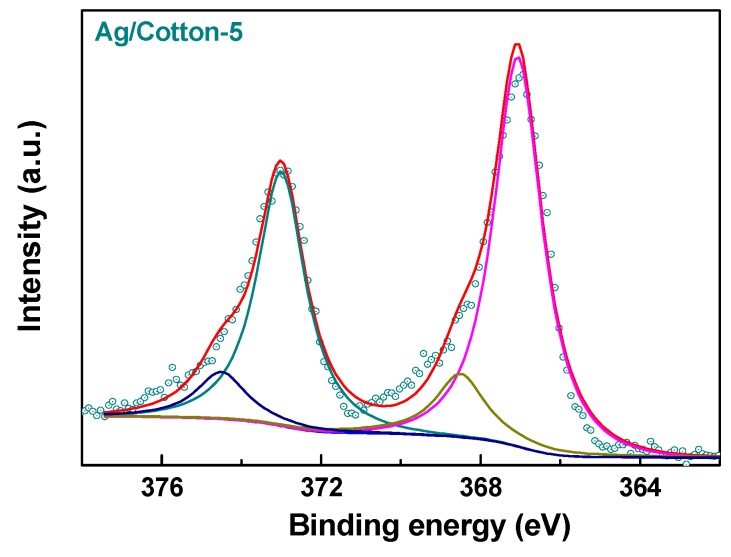
X-ray photoelectron spectroscopy (XPS) spectrum of Ag3d forthe Ag/Cotton-5 sample.

**Figure 4:**
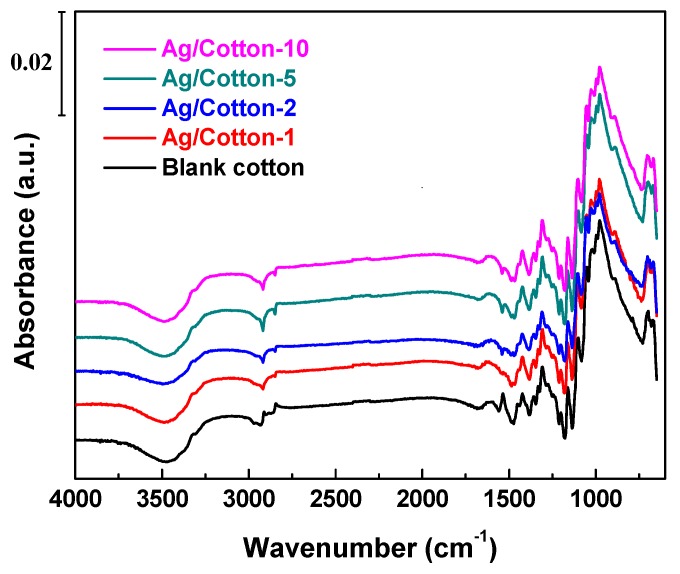
FTIR spectra of the Ag/cotton samples along with the blank cotton.

To further confirm the reduction ability of the surface DBD plasma, an XPS spectrum of Ag3d in Ag/Cotton-5 was measured, as is presented in Figure 3. The Ag3d XPS spectrum can be fitted with two peaks which correspond to metallic silver and Ag(I). According to the XPS data, the composition of metallic silver in the Ag/Cotton-5 sample is as high as 83.3%. This further verifies that surface DBD plasma is an efficient method for reducing silver ions supported on cotton fabric. The incomplete reduction of silver ions may be ascribed to the abundant oxygen-containing functional groups on the cotton fabric surface, which can be identified from the FTIR spectra of the samples (Figure 4). Similar phenomena have been found during the reduction of metal ions supported on activated carbon by plate-to-plate DBD plasma [34].

As shown in Figure 4, the functional groups in the Ag/cotton samples and the blank cotton were identified by FTIR to investigate the effect of the surface DBD plasma treatment. For all the samples, characteristic peaks for cellulose were observed in the range 1000–1200 cm^−1^ [35]. The broad and strong peak band observed at around 3322 cm^−1^ for the samples corresponds to the stretching vibration band of the O-H groups. The medium bands at 2800–3000 cm^−1^ may be ascribed to the symmetric and asymmetric stretching vibrations of the C-H groups in the cellulose samples [36]. The band at 1682 cm^−1^ can be assigned to adsorbed water in the samples. The peaks at 1484 cm^−1^ and 1385 cm^−1^ correspond to the wagging and bending of the C-H functional groups in cellulose, respectively. All of the Ag/cotton samples prepared by surface DBD plasma exhibited similar FTIR spectra to the blank cotton and no new peaks were able to be detected from the FTIR spectra of the Ag/cotton samples. This indicates that surface DBD plasma has no obvious influence on the structure of the cotton fabric support. The nondestructive property of surface DBD plasma is attributed to the fact that the heat-sensitive cotton fabric was not closely contacted with the discharge zone but below it at a 4 mm distance. Hence, the generated active hydrogen species in surface DBD plasma can reduce the silver ions into metallic silver without destroying the cotton fabric.

**Figure 5:**
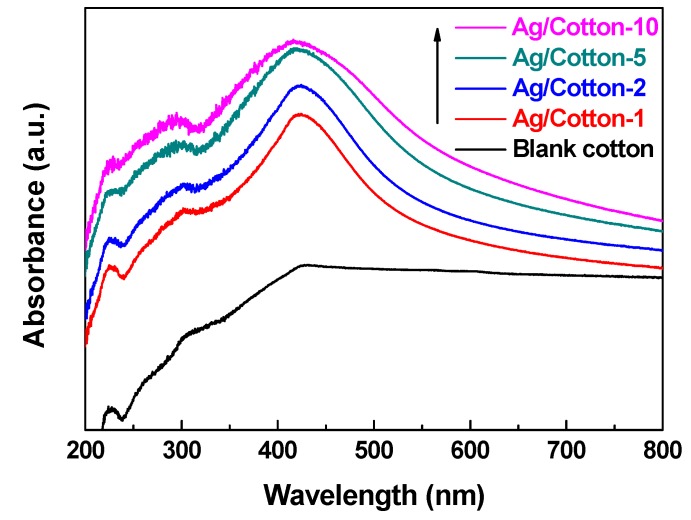
UV-Vis diffuse reflectance spectra (DRS) spectra of the Ag/cotton samples in addition to the blank cotton sample.

Figure 5 shows the UV-Vis DRS spectra of the Ag/cotton samples prepared by surface DBD plasma as well as the blank cotton. Compared to the blank cotton, surface plasmon resonance (SPR) peaks at around 420 nm can be observed for all of the Ag/cotton samples, which further suggests that surface DBD plasma can reduce silver ions into metallic Ag NPs. This is consistent with the XPS result (Figure 3). From Figure 5, we can also see that the intensity of the SPR peak has been enhanced while increasing the loading amount of silver, revealing that more Ag NPs are formed. In addition, the SPR peak becomes broader as the silver loading amount is increased, and redshifts of the SPR peaks are observed for the Ag/cotton samples. The SPR peaks are 415, 419, 423, and 425 nm, respectively, for Ag/Cotton-1, Ag/Cotton-2, Ag/Cotton-5, and Ag/Cotton-10. This is in line with the color change of the samples from light yellow to brownish yellow as seen in Figure 2, demonstrating the size growth of Ag NPs with an increasing silver loading amount. According to the Mie theory, the size of the Ag NPs is in the range 40–70 nm when the SPR peak is located within 415–425 nm [37].

**Figure 6:**
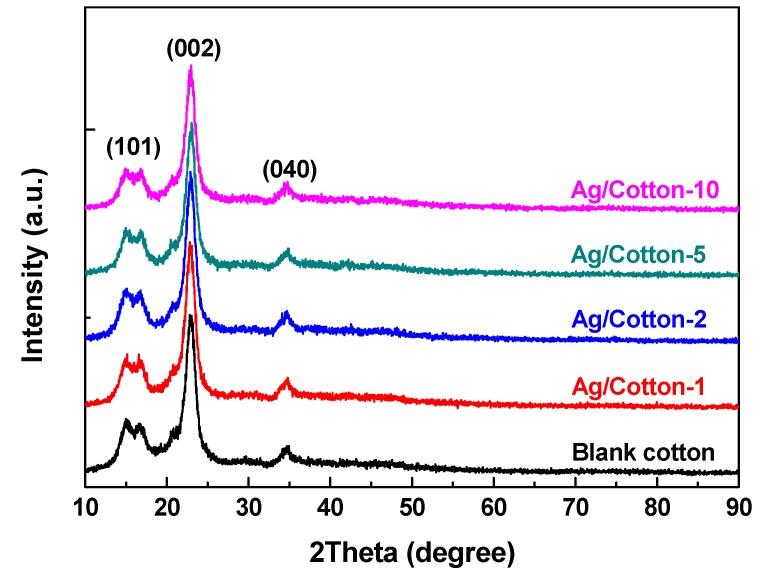
XRD patterns of the Ag/cotton samples as well as the blank cotton sample.

As illustrated in Figure 6, XRD patterns of the Ag/cotton samples and the blank cotton were obtained to further investigate the structure of the samples. For all of the Ag/cotton samples and the blank cotton sample, diffraction peaks at 14.7° and 16.9°were detected, which can be assigned to the (101) plane of cellulose I crystalline form [38]. In addition, characteristic diffraction peaks of cellulose I crystalline at 22.6° and 34.3° corresponding to the (002) and (040) crystal planes are also observed. Obviously, there is no distinct difference among the samples, which further demonstrates that surface DBD plasma did not change the phase structure of the cotton fabric. Interestingly, no characteristic diffraction peak corresponding to silver species was detected for the Ag/cotton samples. A similar phenomenon, which was attributed to the small size of Ag NPs, and the low sensitivity of XRD towards metal detection on organic support, has also been observed in a previous work [39]. It should be noted that the aggregation of Ag NPs as opposed to single Ag NPs results in a color change and SPR absorption peaks broadening for the Ag/cotton samples [40]. This is further supported by SEM and TEM analyses.

**Figure 7:**
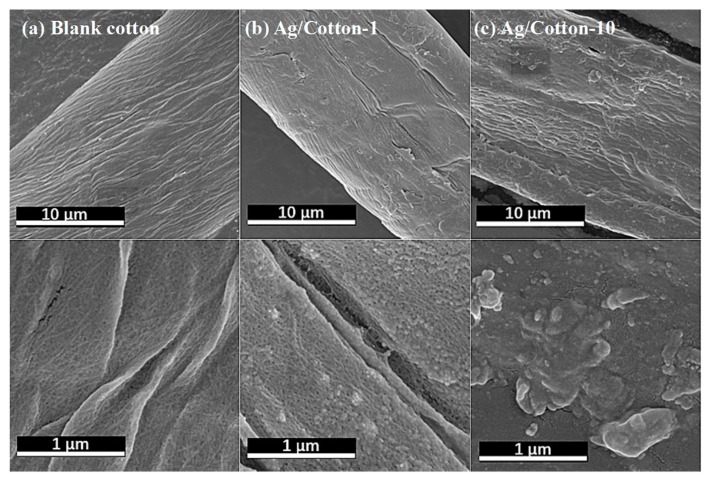
Typical SEM images of (**a**) blank cotton, (**b**) Ag/Cotton-1, and (**c**) Ag/Cotton-10.

To determine the influence of silver deposition on the morphology of the cotton fabric and the distribution of the Ag NPs, typical SEM images of Ag/Cotton-1, Ag/Cotton-10, and the blank cotton sample wereobtained, as shown in Figure 7. The blank cotton without deposition of Ag NPs exhibits a clean and smooth surface. By contrast, the Ag/cotton samples show a rough structure, and aggregated Ag NPs are observed. The size of the Ag NPs is in the tens of nanometers and the size of the Ag NPs tends to grow with an increasing silver loading amount. This is consistent with the UV-Vis DRS results.

**Figure 8:**
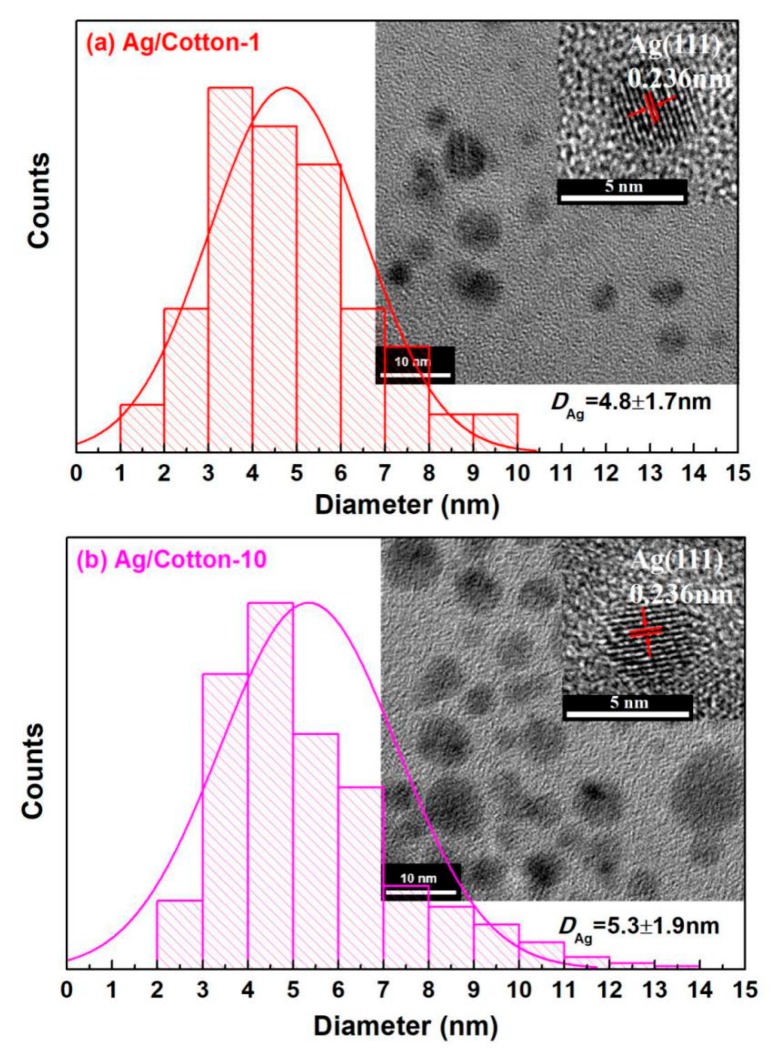
Typical TEM images and histograms of silver nanoparticles (Ag NPs) for (**a**) Ag/Cotton-1 and (**b**) Ag/Cotton-10, and the corresponding high resolution TEM (HRTEM) images of the Ag NPs (insets).

To clearly disclose the distribution of Ag NPs in the Ag/cotton samples, TEM images of Ag/Cotton-1 and Ag/Cotton-10 were obtained, as shown in Figure 8. The Ag NPs in the Ag/cotton samples exhibit a spherical shape are homogeneously distributed. The average size of the Ag NPs (*D*_Ag_) was obtained by measuring more than 300 Ag NPs, and the histograms of size distribution of the AgNPs are also illustrated in Figure 8. *D*_Ag_ in Ag/Cotton-1 and Ag/Cotton-10 were found to be 4.8 ± 1.7 and 5.3 ± 1.9 nm, respectively, demonstrating a slight increase in the size of the Ag NPs at a higher silver loading amount. The size of the Ag NPs detected by TEM was much smaller than that obtained by SEM, indicating the tendency of aggregation of Ag NPs. The Ag NPs were thought to be aggregated after cold plasma reduction due to the hydrophilic property of cellulose [41]. The corresponding HRTEM images of the Ag NPs were also measured, as shown in the insets in Figure 8. Clear lattice fringes with a lattice spacing of 0.236 nm can be observed for the Ag/cotton samples which may be attributed to the face-centered cubic (fcc) Ag (111) planes (*Fm3m*, a = 4.086Å, JCPDS card, file no. 65-2871). This is in line with the XPS and UV-Vis DRS results, revealing that metallic Ag NPs with high crystalline quality are obtained by surface DBD plasma treatment.

**Figure 9:**
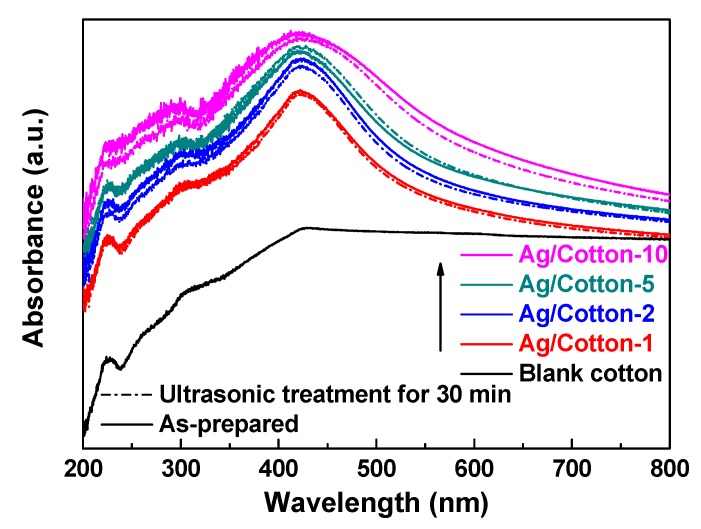
UV-Vis DRS spectra of the Ag/cotton samples as-synthesized and after 30 min of ultrasonic treatment, in addition to the blank cotton sample.

Wash fastness of the Ag/cotton samples was estimated using ultrasonic treatment. UV-Vis DRS spectra of the Ag/cotton samples as-synthesized and after 30 min of ultrasonic treatment are presented in Figure 9. Regarding the UV-Vis DRS spectra of Ag/Cotton-1, Ag/Cotton-2, and Ag/Cotton-5 as-synthesized and after 30 min of ultrasonic treatment, there is no distinct difference that can be observed. However, a slight decrease in the SPR peak intensity may be observed for Ag/Cotton-10 after 30 min ultrasonic treatment due to the high loading amount of silver. These results indicate that surface DBD plasma is a fast and environmentally-friendly method for preparing Ag/cotton samples with a relatively low silver loading amount.

**Figure 10:**
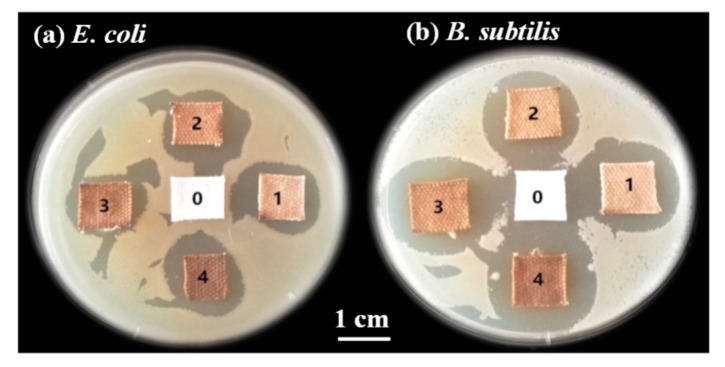
Growth inhibition of (**a**) *E. coli* and (**b**) *B. subtilis* for the samples. 0: blank cotton, 1: Ag/Cotton-1, 2: Ag/Cotton-2, 3: Ag/Cotton-5, 4: Ag/Cotton-10.

The Gram-negative bacterium *E. coli* and Gram-positive bacterium *B. subtilis* with the same concentration were employed to evaluate the antibacterial effect of the Ag/cotton samples, and the results are illustrated in Figure 10. The blank cotton exhibited no inhibition for bacterial growth of *E. coli* and *B. subtilis*. However, obvious bacteriostasis loops can be observed around the Ag/cotton samples with the appearance of *E. coli* or *B. subtilis*. These findings indicate that the surface DBD plasma is efficient for preparing Ag/cotton samples with high antibacterial activity, which may be ascribed to the small size and high distribution of Ag NPs. The average diameters of the bacteriostatic loops for Ag/Cotton-1, Ag/Cotton-2, Ag/Cotton-5, and Ag/Cotton-10 against *E. coli* were found to be 1.38, 1.39, 1.45, and 1.63 cm, respectively. By contrast, the average diameters of the bacteriostatic loops for Ag/Cotton-1, Ag/Cotton-2, Ag/Cotton-5 and Ag/Cotton-10 against *B. subtilis* were 1.85, 1.86, 2.05, and 2.18 cm, respectively. The average diameters of the bacteriostasis loops against both *E. coli* and *B. subtilis* became larger with an increase in the silver loading amount, demonstrating that Ag/cotton samples with a higher silver concentration possess higher antibacterial activity. Moreover, the diameters of the bacteriostasis loops against *B. subtilis* for the same Ag/cotton samples were larger those that against *E. coli*, suggesting that the Ag/cotton samples exhibit higher antibacterial activity against Gram-positive bacterium *B. subtilis* than Gram-negative bacterium *E. coli*, which is in line with the results of Li et al. [25]. This may be due to the structure of the cell wall of the bacteria [42].

## 4. Conclusions

Cotton-fabric-supported Ag NPs (Ag/cotton) have been successfully prepared in this work using an environmentally-friendly and fast surface DBD plasma method for the first time. No additional reducing chemicals except the active hydrogen species generated in cold plasma served as the reducing agents. The color of the samples changed from colorless to yellow after surface DBD plasma treatment and the color changed from light yellow to brownish yellow with an increase in the loading amount of silver. Moreover, SPR peaks and the Ag3d XPS results further confirm that the Ag species mainly exist in the metallic state. TEM images show that the Ag NPs are uniformly distributed on the fiber surface and that the size of the Ag NPs is in the range 4.8 to 5.3 nm. FTIR and XRD results demonstrate that heat-sensitive cotton fabrics are not destroyed by surface DBD plasma, which may be due to the fact that the cotton fabric is not in close contact with the discharge zone during the treatment but below it at a 4 mm distance. Wash fastness estimated using ultrasonic treatment revealed that the Ag NPs in the Ag/cotton samples possess high adhesion ability to the cotton fabric. The Ag/cotton samples exhibit high antibacterial activity for both the Gram-negative bacterium *E. coli* and the Gram-positive bacterium *B. subtilis*. This work provides a universal, fast, simple, and environmentally-friendly cold plasma method for synthesizing Ag NPs on heat-sensitive materials at atmospheric pressure.
